# Malicious Mites—*Sarcoptes scabiei* in Raccoon Dogs (*Nyctereutes procyonoides*) in Schleswig-Holstein, Germany

**DOI:** 10.3390/pathogens12121379

**Published:** 2023-11-22

**Authors:** Jana C. Klink, Alexandra Rieger, Hermann Ansorge, Sophie Aurich, Christiane Hoffmann, Christa Ewers, Marie-Kristin Raulf, Christina Strube, Ursula Siebert

**Affiliations:** 1 Institute for Terrestrial and Aquatic Wildlife Research, University of Veterinary Medicine Hannover, Foundation, 30559 Hannover, Germany; jana.christina.klink@tiho-hannover.de (J.C.K.); alexandra.rieger@tiho-hannover.de (A.R.); 2 Senckenberg Museum of Natural History Görlitz, 02826 Görlitz, Germany; hermann.ansorge@senckenberg.de; 3 International Institute Zittau, Technische Universität Dresden, 02763 Zittau, Germany; 4 Institute for Hygiene and Infectious Diseases of Animals, Justus Liebig University Giessen, 35392 Giessen, Germany; sophie.aurich@vetmed.uni-giessen.de (S.A.); christiane.hoffmann@vetmed.uni-giessen.de (C.H.); christa.ewers@vetmed.uni-giessen.de (C.E.); 5 Institute for Parasitology, Centre for Infection Medicine, University of Veterinary Medicine Hannover, Foundation, 30559 Hannover, Germany; marie-kristin.raulf@tiho-hannover.de (M.-K.R.); christina.strube@tiho-hannover.de (C.S.)

**Keywords:** *Sarcoptes scabiei*, mange, raccoon dog, invasive species, alopecia, hyperkeratosis, scanning electron microscopy

## Abstract

Sarcoptic mange was detected in five free-ranging raccoon dogs (*Nyctereutes procyonoides*) in the federal state of Schleswig-Holstein, Germany, during a health assessment study of invasive species, including raccoon dogs, carried out between 2021 and 2022. Four raccoon dogs showed severe lesions, including extensive alopecia with thickening and hyperpigmentation of the skin (lichenification). The fifth animal was less affected, showing only thinning of the hair coat in multiple body locations. Skin scrapings were performed and confirmed the presence of *Sarcoptes scabiei.* Histopathology of the skin revealed diffuse epidermal hyperplasia and hyperkeratosis, mild eosinophilic dermatitis, and varying amounts of intralesional mites. *Staphylococcus pseudintermedius* and *Corynebacterium auriscanis* were detected in the skin samples of the affected animals, indicating a secondary bacterial infection. The source of sarcoptic mange remains unclear; interspecies transmission via direct or indirect contact seems likely. Raccoon dogs are therefore a potential vector for sarcoptic mange, and their behaviour could contribute to disease spread and persistence.

## 1. Introduction

The raccoon dog (*Nyctereutes procyonoides*) is an invasive alien species originating from East Asia that was introduced to the former Soviet Union between 1928 and 1956 [1,2]. Since then, it has successfully conquered large parts of North and Central Europe [3,4]. In the German federal state of Schleswig-Holstein, the raccoon dog has been reproducing since the 1990s [5]. The neozoan species is described as a potential predator of and competitor for native species as well as a reservoir and vector for infectious diseases [3,6,7,8]. For example, in Finland, raccoon dog colonisation is correlated with the re-emergence of rabies [9,10].

Sarcoptic mange is an ectoparasite infestation of the skin caused by the mite *Sarcoptes scabiei,* which can affect a wide range of hosts, including domestic and wild animals as well as humans [11,12]. The raccoon dog, along with other canid species such as the native red fox (*Vulpes vulpes*) and the domestic dog (*Canis lupus familiaris*), can also become infested [3,6,11]. 

*S. scabiei* forms a single species [11,13], being divided into varieties specific to different hosts, for example, *S. scabiei* var. *canis*, var. *vulpes*, var. *hominis*, etc., being physiologically specialised and only distinguishable by molecular analysis [11,12]. Nevertheless, host specifications are varying, and the extends are still much discussed [12]. Interspecies transmission-bearing zoonotic potential has been described [12,14,15]. Disease transmission is possible by direct or indirect contact, as mites can survive for a few days without a host and remain infectious [12,13]. Raccoon dogs dig burrows themselves but often use old fox and badger dens for shelter, reproduction, and hibernation [16,17]. This, as well as their dispersal behaviour [10,18,19], might favour the spread of sarcoptic mange and the interspecies transmission rate.

To the authors’ knowledge, literature on detailed examinations of raccoon dogs with sarcoptic mange in Europe is lacking. Previous studies diagnosed sarcoptic mange based on observation of skin alterations but not thorough diagnostics [19,20,21]. However, several publications on pathological findings in infested raccoon dogs in Japan and Korea exist [22,23,24,25,26,27,28,29]. 

The aim of this case series is to give a detailed overview of pathological findings in raccoon dogs in the German federal state of Schleswig-Holstein suffering from sarcoptic mange, compare findings to other affected species and the available literature, and discuss the raccoon dogs’ role in disease transmission and spreading.

## 2. Materials and Methods

### 2.1. Animals, Postmortem Examination, and Sample Preparation

**Figure 1:**
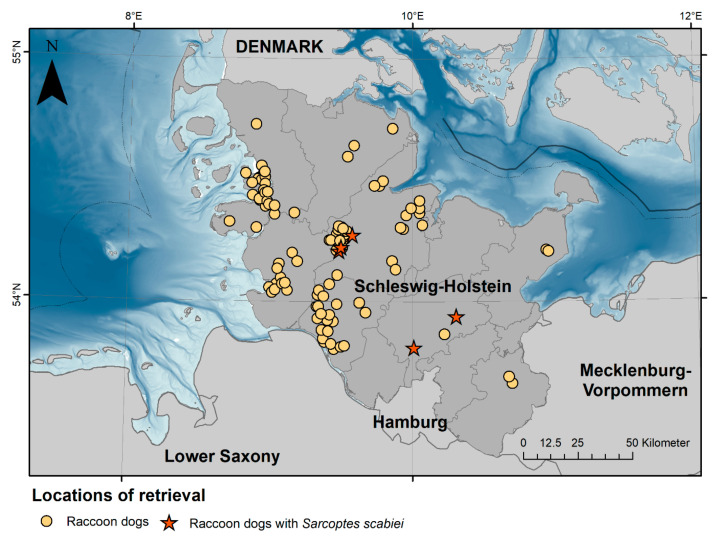
Locations of retrieval of the 110 raccoon dogs (*Nyctereutes procyonoides*) investigated in the federal state of Schleswig-Holstein, Germany. Raccoon dogs infested with sarcoptic mange are presented as red stars in the map.

In the years 2021 and 2022, a health and risk assessment study of selected invasive species, including raccoon dogs, was conducted in the federal state of Schleswig-Holstein, Germany. In total, 110 raccoon dogs were investigated (Figure 1). The animals that were either shot or found dead underwent a comprehensive postmortem examination at the Institute for Terrestrial and Aquatic Wildlife Research (ITAW) of the University of Veterinary Medicine Hannover following a modified necropsy protocol, as described by Fähndrich et al. [30]. Selected biological data were collected, including bodyweight, standardised measurements of total length, snout-vent length, tail length, hindfoot length and ear length, sex, and nutritional status. Nutritional status (Supplements Table S2) was scored as “good”, “moderate”, or “poor”, depending on the amount of subcutaneous and retroperitoneal fat, the development of the skeletal musculature, and age. Animals scored as “good” showed a thick layer of subcutaneous and retroperitoneal fat with well-developed musculature, while a poor nutritional status was given if subcutaneous and organ fat were absent with musculature eventually showing signs of atrophy. The score of “moderate” included animals not matching the other categories. In addition, season was taken into account, as raccoon dogs do show seasonal obesity in regions with cold winters to survive periods of food shortages [31]. However, juveniles in the process of building up fat reserves could not be assessed regarding their nutritional status. All animals were divided into two age classes (juvenile and adult) by size, teeth (deciduous or permanent), and tooth wear. The carcasses were investigated for external lesions, and all organs were examined macroscopically. Images of the corpses and of detected lesions were taken with a digital camera (Panasonic, Model No. DMC-TZ101). Five animals showed skin lesions that could have been attributed to sarcoptic mange; three of those were fresh, while two were frozen prior to necropsy. Standardized samples and additional samples of relevant lesions were collected for parasitological, light and electron microscopy, microbiological analyses, and age determination.

### 2.2. Parasitology

In all raccoon dogs with suspected sarcoptic mange, skin samples were taken and stored at −20 °C until further examination. Skin scrapings of thawed samples were performed using a scalpel blade scraping of the upper skin layers in the transition zones between lesions and unaffected or lesser affected areas. Samples were incubated in 10% KOH for 1 h at 37 °C to digest non-chitinous material such as skin. Isolated mites were separated from digested tissue by sedimentation with subsequent flotation in a 50% sucrose solution and examined microscopically (Primostar 1; Carl Zeiss Microscopy Deutschland GmbH). Species identification of *S. scabiei* was based on typical morphological characteristics such as the characteristic oval, ventrally flattening, and dorsally convex tortoise-like body, stout dorsal setae, numerous cuticular spines, and ventrally ridged cuticular striations [32]. Furthermore, the presence of long, unsegmented stalk-like pretarsi on legs I served as an identification characteristic. Delineation to *Notoedres cati* was based on the location of the anal opening (terminal for *S. scabiei*, dorsal for *N. cati*) and the presence of dorsal spines (present for *S. scabiei*, absent for *N. cati*) [33].

### 2.3. Histopathology and Scanning Electron Microscopy (SEM)

Depending on the state of decomposition, samples of all major organ systems were taken and fixed in 10% buffered formalin. Subsequently, the samples were embedded in paraffin wax, sectioned at 3 µm, and stained with hematoxylin and eosin (HE).

For SEM, formalin-fixed samples of the skin of one animal were trimmed and post-fixed for 48 h in a glutaraldehyde solution (2.5% in Sorensen’s phosphate buffer). Dehydration of specimens was subsequently achieved via a graded series of acetone (50%, 60%, 70%, 80%, 90%, and 100%). Using a carbon dioxide (CO_2_) drying tool (Union Point Dryer CPD 030, BAL-TEC AG, Balzers, Liechtenstein), critical point drying (CPD) was carried out with the following fixation of samples on aluminium plates that were covered with conductive carbon pads (Leit-Tabs, Plano, Marburg, Germany) and coated by a sputter coater ( Union SCD 040, BAL-TEC, Balzers, Liechtenstein) with a gold/palladium layer of 12 nm thickness. SEM imaging was carried out using a Zeiss Digital Scanning Microscope (DSM 950, Oberkochen, Germany). The brightness and contrast of images were adjusted in Adobe (San Jose, CA, USA) Photoshop^®^ 2023.

### 2.4. Microbiology

For all five animals, samples of skin, lung, liver, spleen, kidney, and small and large intestines were taken for microbiological examination. Additional organs were also examined in each case from individual animals; a detailed list can be found in Supplements Table S1. Samples were stored at −20 °C until cultivation at the Institute of Hygiene and Infectious Diseases of Animals, Justus Liebig University, Giessen, Germany. Thawed tissue samples were sterilised by flame and sectioned using sterile scissors. Each sample was cultured on blood agar (Oxoid, Wesel, Germany) containing 5% defibrinated sheep blood and incubated for 48 h at 37 °C in ambient air.

Bacterial growth was estimated semi-quantitatively by the number of colonies observed. Approximately 1–5 colonies were rated as (+) for isolated bacterial growth, 6–50 colonies as + for low bacterial growth, 51–200 colonies as ++ for moderate bacterial growth, and > 200 colonies as +++ for strong bacterial growth.

Pure cultures of grown colonies were identified with the direct smear technique using MALDI TOF-MS (Biotyper microflex LT/SH, Bruker Daltonics, Bremen, Germany) with the MBT Compass Library (v10.0.0.0, 9607 MSP; Bruker Daltonics) according to the manufacturer’s instructions.

## 3. Results

### 3.1. Gross Pathology

**Figure 2:**
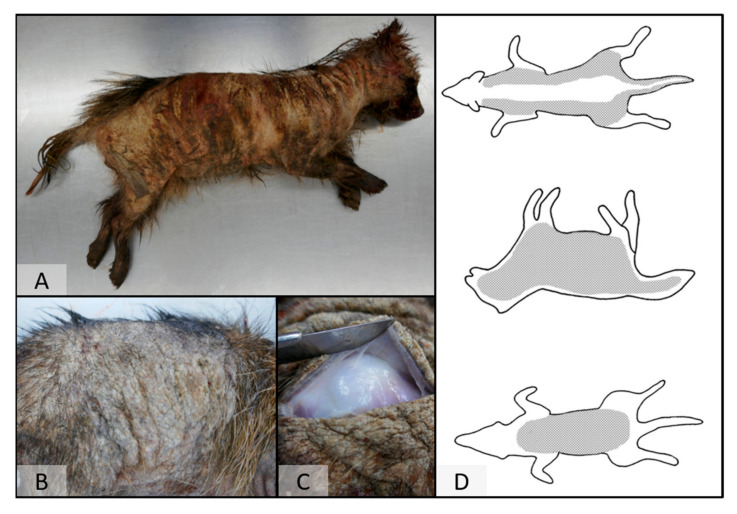
The raccoon dog (*Nyctereutes procynoides*) is severely affected by sarcoptic mange. The animal showed diffuse alopecia (**A**), severely thickened and hyperpigmented skin, as well as moist crusts (**B**,**C**). Schematic depiction of the distribution pattern of skin lesions in a severely infested animal (**D**).

**Figure 3:**
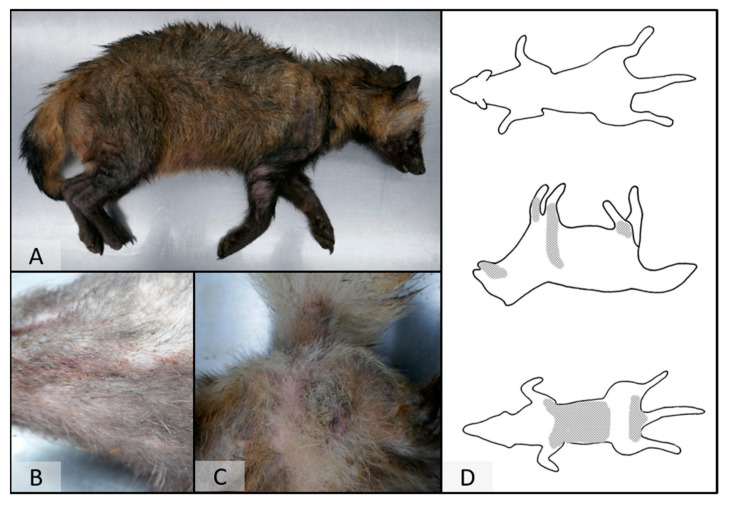
The raccoon dog (*Nyctereutes procynoides*) is mildly affected by sarcoptic mange. Skin lesions were present on the lateral hind limbs, extending the lateral body towards the abdomen (**A**–**C**). Schematic depiction of the distribution pattern of skin lesions in a mildly infested animal (**D**).

On post-mortem examination, four raccoon dogs showed marked alopecia with a distinct distribution (Figure 2). The skin was severely thickened and hyperpigmented, as well as partially covered by moist crusts. In the most severely affected animal, skin alterations covered almost the whole body. In less affected cases, lesions were present on the lateral hind limbs, extending the lateral body towards the abdomen (Figure 3). The fifth animal presented with only mild skin alterations consisting of hair loss of the limbs, especially on the lateral sites, as well as at the abdominal region, the thighs, and the ventral part of the root of the tail (Figure 3). Pence et al. [34] described four classes of progression of lesions of sarcoptic mange in coyotes from Texas. The distribution pattern in this species differs from the one in raccoon dogs; however, the less affected animal comprises class I (initial lesions) and the severely affected raccoon dogs class III (more than half of the total body surface affected) lesions [12,34].

### 3.2. Parasitology

**Figure 4:**
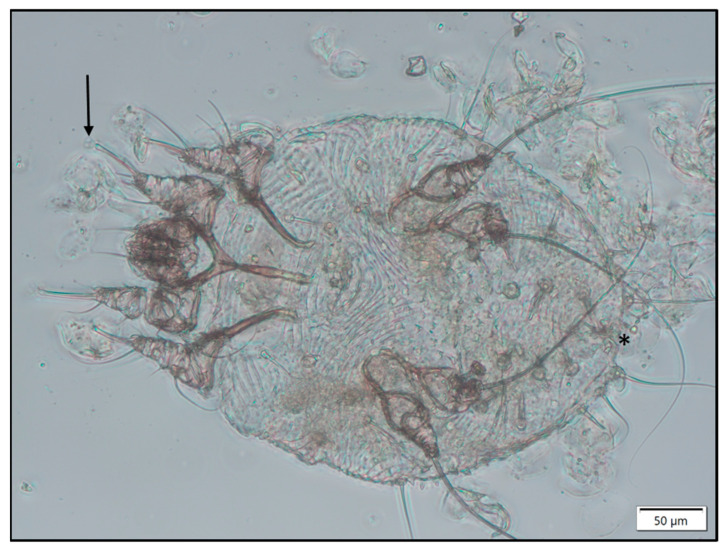
*S. scabiei* is characterised by its oval, tortoise-like body, numerous cuticular spines, ventrally ridged cuticular striations, long unsegmented stalk-like pretarsi on legs I (see arrow), and the terminal anal opening (see asterisk).

In all skin scrapings performed, *Sarcoptes* mites were diagnosed based on typical morphological characteristics (Figure 4).

### 3.3. Histopathology and Scanning Electron Microscopy (SEM)

**Figure 5:**
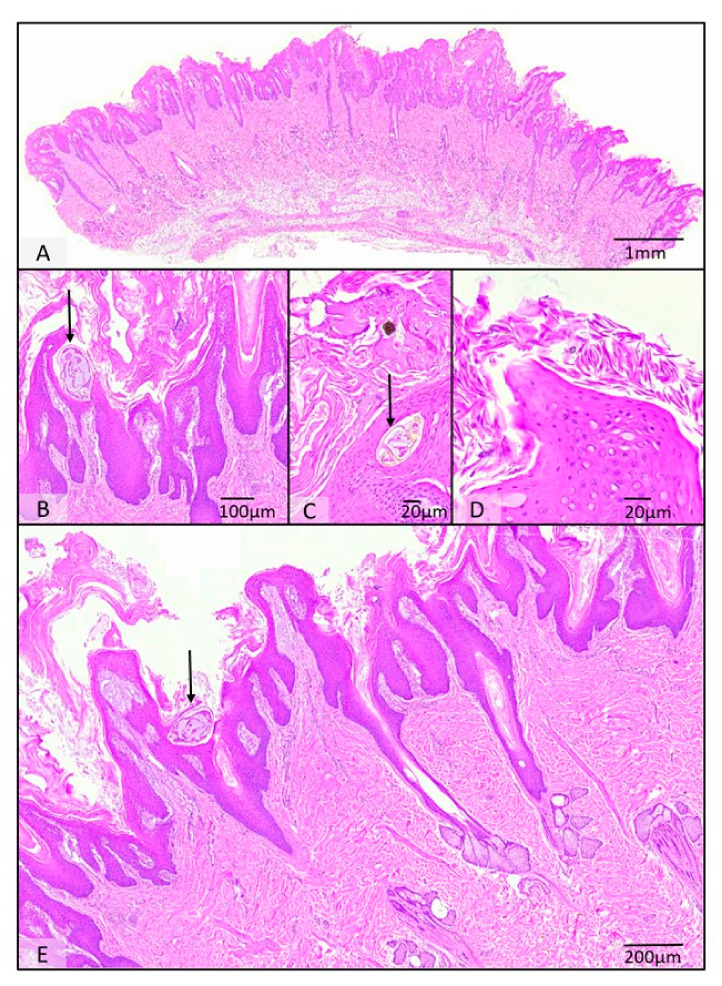
Histopathological findings of the skin of severely affected raccoon dogs *(Nyctereutes procynoides*). There was diffuse thickening of the epidermis (**A**), characterised by a severe pseudocarcinomatous hyperplasia of the epidermis (**A**,**E**), as well as a prominent hyperkeratotic ortho- and parakeratotic hyperkeratosis (**B**–**E**). In multiple locations, there are purulent serocellular crusts, which contain multifocal bacteria (**D**). Intralesional mites (marked with arrows) measuring 150 × 200 µm could be identified (**B**,**C**,**E**). A mild to moderate subepidermal oedema is present (**D**), as well as a hair follicle atrophy (**E**).

Histology of the skin of all five animals with confirmed sarcoptic mange yielded an overall prominent ortho- and parakeratotic hyperkeratosis with varying degrees of epidermal hyperplasia ranging from mildly irregular to pseudocarcinomatous. Within epidermal burrows, single to numerous *Sarcoptes* mites of up to 200 µm in diameter were found and characterised by their chitinous exoskeleton with dorsal spines, short-jointed limbs, striated musculature, and occasionally visible digestive and reproductive tracts. Dermal changes comprised mild to moderate multifocal subepithelial oedema and an associated mixed cellular infiltration comprised of mostly eosinophils as well as lymphocytes, plasma cells, and histocytes, with occasional aggregates of mast cells (Figure 5).

Hair follicles are mostly presented without hair shafts, occasionally with increased trichilemmal keratinization. Sebaceous glands were often atrophic. Multifocally, serocellular crusts with intralesional coccoid bacteria were seen (Figure 5).

Additionally, two animals displayed interstitial pneumonia rich in eosinophils and associated with prominent or hypertrophic airway smooth muscle. Three animals also showed mild to moderate suppurative bronchopneumonia, in one case also associated with airway smooth muscle hypertrophy and in another case with eosinophils present (Supplements Table S2).

Reactive hyperplasia of several lymph nodes, including retropharyngeal, pulmonary, mesenterial, aortic, and inguinal lymph nodes, was a major finding in all animals. Two raccoon dogs also presented hyperplasia of the splenic white and red pulp; one showed follicular hyperplasia of the spleen, and two animals showed trilinear hyperplasia of the bone marrow. Other secondary findings included mild hepatolipidosis; for detailed information, see Supplements Table S2.

**Figure 6:**
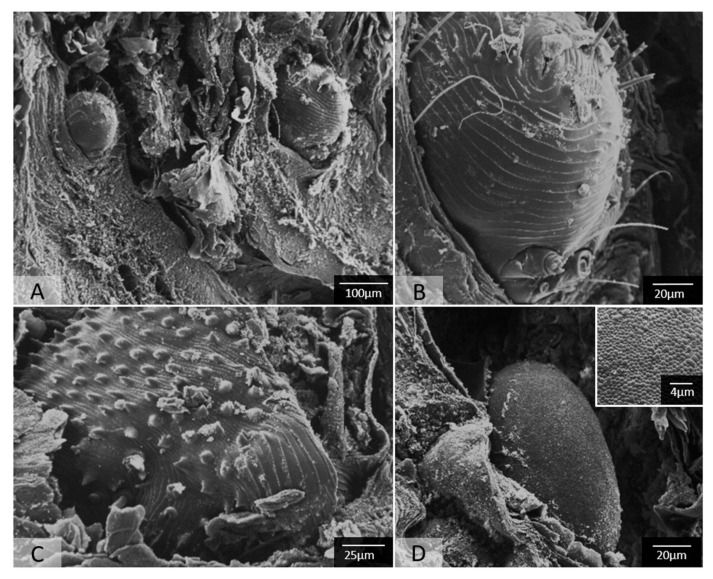
SEM findings of the skin of *S. scabiei*-infested raccoon dogs *(Nyctereutes procynoides*). Sarcopes mites live in tunnels that they burrow into the keratin layer of the epidermis (**A**). Adult mites display ventrally ridged striations and a prominent terminal anal opening (**B**), as well as a cuticula with numerous spines (**C**). Within the burrows, ellipsoid eggs are also found (**D**), displaying a reticulate surface structure (see inset). Images are courtesy of the Institute of Veterinary Pathology, LMU Munich.

Scanning electron microscopy nicely revealed the intracorneal burrows of the mites, their aforementioned characteristics including ventrally ridged striations, cuticular spines, and a terminal anal opening, as well as *Sarcoptes* mite eggs that possess a peculiar slightly uneven reticulate surface pattern (Figure 6).

### 3.4. Microbiology

In total, we identified 24 different species in the examined organs (Supplements Table S1). The most frequently detected bacterium was *Staphylococcus pseudintermedius*, followed by coagulase-negative *staphylococci*, *α-streptococci*, *Corynebacterium auriscanis*, and β-haemolytic *Streptococcus canis*. Especially *Staphylococcus pseudintermedius* was distributed with considerable growth in nearly every organ of all five animals. *Streptococcus canis* was predominantly found in the organs of animals Np3 and Np5, and *Corynebacterium auriscanis* was mainly present in the organs of animals Np3 and Np5 as well. In the skin, *Staphylococcus pseudintermedius* was detected in strong or moderate growth in all five animals. In three animals, it was found in combination with *Corynebacterium auriscanis* in skin samples.

## 4. Discussion

This case series outlines the pathological, microbiological, and ectoparasitological findings in raccoon dogs with sarcoptic mange in Northern Germany.

Macroscopical skin lesions, as well as the distribution pattern and histopathological findings, are similar to those described in raccoon dogs in Korea and Japan [23,24,25,26,27].

While the examined raccoon dogs mostly showed skin lesions reaching from the lateral hind limbs via the lateral site of the body towards the abdomen, a somewhat different distribution pattern is described in other canid species. The initial lesions in foxes start from the distal limbs and the ischium, and as the infestation progresses, the animals show partial or diffuse alopecia of the limbs, lateral site of the body, abdomen, chest, shoulders, tail, and head [12]. In dogs, the initial lesions start on the head and pinna of the ear and extend from there to the ventral thorax, abdomen, and legs, affecting especially the elbow and hock. The back is normally not infested, but if the disease occurs generalised, the whole body is affected [11,12,35]. It is described that *S. scabiei* selects favoured areas because of the lipid composition and other site-specific factors of the skin [36,37], which could explain the varying distribution patterns in different species.

*S. scabiei* can modulate the innate and adaptive immune responses of its host; those abilities have been identified as anti-inflammatory, anti-immune, and anti-complement activities [36]. In early infestation, these abilities may result in a delayed immune and inflammatory response that allows mites to establish [36]. Once the mite population reaches a certain threshold, the delay might be overridden, and the inflammatory reaction occurs [36]. The observed alterations indicate that the infested animals might not have been able to evoke an immune response sufficient enough to control the dissemination of mites [12]. In immunologically competent hosts, the formation of fibroplasia and chronic inflammation of the dermis, as well as large numbers of eosinophils in the dermis and epidermis and rare to absent mites, are described [12].

Reactive hyperplasia of lymph nodes and tonsils and hyperplasia of the splenic white and red pulp could be interpreted as a direct response to the local inflammation of the skin as well as to the ongoing immune defence process.

All of the examined raccoon dogs confirmed with sarcoptic mange presented pneumonia. In some cases, this is associated with eosinophils and hypertrophic airway smooth muscle. Eosinophils in general are mainly associated with inflammatory reactions to parasites or allergic responses [38]. Since no lung parasites could be identified in any of the animals examined, the origin of eosinophils in the pulmonary interstitium might represent a reaction to the haematogenous circulation of immune complexes or antigens. Smooth muscle hypertrophy is a finding that has been described in humans with asthma [39,40]. Being a component of remodelling, this alteration can occur in chronic, long-standing disease [41]. The suppurative bronchopneumonia might be caused by a secondary bacterial infection. In a study conducted in Japan, in 14 out of 43 raccoon dogs with sarcoptic mange, varying types of pneumonia were the underlying cause of death [26]. However, it is not mentioned how many animals actually had pneumonia, as in this study the underlying cause of death is presented and not pathological findings in general. Therefore, even more animals could present with pneumonia. It is unclear whether the pneumonia was initially affecting the raccoon dogs, making the animals more prone to an infestation with *S. scabiei*, or if the sarcoptic mange favours pneumonia due to the circulation of immune complexes and antigens. However, the observed concurrent presence of sarcoptic mange and pneumonia should not be overestimated, as only five mange-affected animals were available in the present study.

Skin infections with *Streptococcus canis* in dogs are described as sporadic and opportunistic, and *Staphylococcus pseudintermedius* can also be isolated from the skin of healthy dogs [42,43]. Coryneform bacteria are described as part of the normal flora of the dog skin [44]. *Corynebacterium auriscanis* is a potential opportunistic pathogen present in mixed infections in Otitis externa in dogs [44]. It has the capability to cause lesions and clinical infections by itself but should not be viewed as a primary pathogen in canine dermatitis [45]. In the examined raccoon dogs, *Streptococcus canis, Staphylococcus pseudintermedius,* and *Corynebacterium auriscanis* have been cultivated from different organs; the latter two have also been detected in the skin. These pathogens are associated with pyoderma and otitis and pose a zoonotic risk [46,47,48,49]. *S. scabiei* itself might contribute to the spread of pathogenic bacteria, as streptococci and staphylococci have been isolated from skin burrows and mite faeces [26,50]. Additionally, it is discussed that the mite inhibits complement activity and therefore promotes bacterial colonisation [36]. In a breeding farm in China, a mass outbreak of *Staphylococcus pseudintermedius* was described, causing severe skin and soft tissue infections as well as dyspnea and pathological lesions in other organs [51]. However, since the affected animals were kept in captivity, the stocking density made disease transmission more likely than in free-living animals. Rather than posing a risk for healthy animals or humans, the described pathogens are more likely to be an issue for animals with pre-existing skin lesions, including the self-trauma-induced excoriations in sarcoptic mange. Moderate to high growth of *Staphyloccoccus pseudintermedius* was present in the skin of all five raccoon dogs, while moderate to high growth of *Corynebacterium auriscanis* was present in the skin of three raccoon dogs (Np2, Np3, Np5), both bacterial species indicating a secondary bacterial infection. Np5 being the animal with less pronounced skin alterations, also shows a high growth of *Staphylococcus pseudintermedius* and a moderate growth of *Corynebacterium auriscanis*, indicating that the severity of bacterial colonisation might not be correlated with the morphological manifestation of the skin alterations. There is a high risk for secondary infection, with concomitant immunosuppression facilitating spread and subsequent septicaemia as a potential fatal outcome. Though bacteria were found on numerous organs, gross and histopathologic examinations did not reveal alterations in internal organs associated with sepsis. Instead of haematogenous dissemination of bacteria *ante mortem*, the distribution might be caused by the advanced decomposition state of the animals and bacterial translocation. On the other hand, three raccoon dogs (Np1, Np3+Np4) presented suppurative bronchopneumonia; in two cases (Np1+Np3), *Staphylococcus pseudintermedius* and *Streptococcus canis* with low to strong bacterial growth were found, indicating a secondary infection of the lung associated with the infection of the skin. The described pathogens are potentially zoonotic and can lead to severe infections in single animals, or, in the case of *Staphylococcus pseudintermedius*, have the ability to cause mass outbreaks under certain conditions in raccoon dogs [51].

*S. scabiei* var. *nyctereutis,* a raccoon dog-specific variety sharing 99% identity with the mitochondrial genome sequences of *S. scabiei* var. *canis*, var. *hominis*, and var. *suis*, was recently described in raccoon dogs from Japan [52]. A study of mange in raccoons in Germany suggests a fox origin of the *Sarcoptes* mite, showing that pathogens can be shared among populations of native and invasive carnivores [53]. In a study in Sweden, mites from foxes and raccoon dogs formed one compact cluster based on microsatellite genotyping [54], which indicates that raccoon dogs are infested with *S. scabiei* var. *vulpes* in this region rather than with a species-specific variety. To the authors’ knowledge, *S. scabiei* var. *nyctereutis* has not been encountered in Europe yet. In one of the two locations in Schleswig-Holstein where the raccoon dogs with confirmed sarcoptic mange were found, red foxes (*Vulpes vulpes*) and badgers *(Meles meles)* were also reported to show clinical signs that could be attributed to sarcoptic mange. The observation of other carnivore species that might be infested with *S. scabiei* in the same location indicates an interspecies transmission. Raccoon dogs often inhabit old fox and badger setts [16,17,55], this could be a possible transmission route. Nevertheless, the origin of the infestation is unclear, and even if *S. scabiei* var. *nyctereutis* has not been reported in Europe yet, the determination of the variety is necessary to allow a final answer.

Generally, sarcoptic mange has no effect on long-term population dynamics but can be an issue in fragmented or remnant populations of threatened species [12]. It has been described as an important mortality factor in raccoon dogs and has caused temporary and even significant declines in red fox populations [56,57,58]. In the Netherlands, a higher mortality of foxes caused by sarcoptic mange is assumed since the colonisation of the raccoon dog [3,59]. In addition, a high population density might be a factor in the epizootics of sarcoptic mange; the infestation might persist in the population and might have an influence on changed population densities [22,28,60]. In the hunting period 2021/22 (1 April 2021–31 March 2022), the hunting bag in Schleswig-Holstein consisted of 9942 raccoon dogs [61], indicating a high population density, which might increase the risk of disease spread. In our study, five raccoon dogs were diagnosed with sarcoptic mange, making up 4.5% of the examined animals. The sample size does not allow conclusions on the prevalence of sarcoptic mange in Schleswig-Holstein, as it does not reflect the entire population and might be biased by the sampling technique (e.g., animals showing symptoms might be reported more often, motivation of hunters, shot vs. deceased animals, skin scrapings only being performed in animals with skin lesions, etc.).

In conclusion, *S. scabiei*, a potentially zoonotic pathogen, infests raccoon dogs in Schleswig-Holstein, Germany. Although the transmission route is unclear and further determination of the variety of the mite is necessary, the presence of other possibly infested animal species, such as foxes and bagders, in the same regions could indicate an interspecies transmission. Raccoon dogs can act as reservoirs and vectors for various infectious diseases, including sarcoptic mange, and their behaviour can contribute to disease spread and persistence. Long-term health monitoring of this alien species, including surveillance of various infectious diseases to evaluate their potential risk for human and animal health, is necessary.

## Data Availability

The data presented in this study are available in this published article and its supplementary information files.

## Supplementary Materials

The following supporting information can be downloaded at: https://www.mdpi.com/article/10.3390/pathogens12121379/s1; Table S1: Detected bacterial and fungal microorganisms in raccoon dogs presented with sarcoptic mange; Table S2: Biological data and pathological findings of the five investigated raccoon dogs presented with sarcoptic mange.
