# Supplementary material for: Malicious Mites—Sarcoptes scabiei in Raccoon Dogs (Nyctereutes procyonoides) in Schleswig-Holstein, Germany
Source: Pathogens. 2023 Nov 22;12(12):1379. doi: 10.3390/pathogens12121379 (PMC10745952; doi:10.3390/pathogens12121379)
Supplement: Supplementary file 1 [file pathogens-12-01379-s001.zip › pathogens-2653210-supplementary.pdf]

Supplements

Table S1: Detected bacterial and fungal microorganisms in raccoon dogs presented with sarcoptic mange

Animal	Organ \ Microorganism	No growth	*Acinetobacter* spp.	aerobic bacilli	α-streptococci	*C. perfringens*[1]	*Coryne. auriscanis*[2]	*Enterobacter* spp.	*Enteroc. faecalis*[3]	*Escherichia coli*	*Fungi*[4]	*Lactococcus* spp.	*Macrococcus* spp.	other *Enterobacterales*[5]	*Pseudomonas* spp.	*Psychrobacter* spp.	*S. pseudintermedius*[6]	Coag.-neg. staph.[7]	*Strep. canis*[8]	*Strep. dysgalactiae*	*Vagococcus* spp.
Raccoon dog 1 (Np1)	Liver			(+)	+			+				+		+			+	+			
	Spleen											(+)					+	+			
	Kidney				+									(+)			++	+			
	Lung		+		+				+			+		+			+++	+	+		
	Small Intestine			+	++	+++						+		(+)			++				
	Large Intestine			++	++	+++						+					+				
	Brain				(+)							(+)		(+)			+	+			
	Reproductive Organs	+																			
	Skin													+		+	+++				
Raccoon dog 2 (Np2)	Liver												+				(+)				
	Spleen																				
	Kidney				+		+						+	(+)			+	++		+	
	Lung				+		+						+				+	+			
	Small Intestine				+		+						+				(+)	+			
	Large Intestine				+		+			+			+				(+)	+			
	Mesenteric lymph node						+						+				(+)	+			
	Brain				+		++						++				+	++		+	
	Reproductive Organs				+		+						+				+	++			
	Skin						+++						+				+++	+++			

(continued)

Animal	Organ \ Microorganism	No growth	*Acinetobacter* spp.	aerobic bacilli	α-streptococci	*C. perfringens*[1]	*Coryne. auriscanis*[2]	*Enterobacter* spp.	*Enteroc. faecalis*[3]	*Escherichia coli*	*Fungi*[4]	*Lactococcus* spp.	*Macrococcus* spp.	other *Enterobacterales*[5]	*Pseudomonas* spp.	*Psychrobacter* spp.	*S. pseudintermedius*[6]	Coag.-neg. staph.[7]	*Strep. canis*[8]	*Strep. dysgalactiae*	*Vagococcus* spp.
Raccoon dog 3 (Np3)	Liver													(+)		+			+		
	Spleen				+											+	(+)	(+)			
	Kidney													(+)	+	+	+	+	++		
	Lung			+	+									+	+	++	+	++	++		
	Small Intestine			+	+	+											+	+	+		
	Large Intestine			+	+	+++									+		+	+	+		
	Mesenteric lymph node				+												+	+	+++		
	Brain				+++									+	+		++	++	+		
	Reproductive Organs				+									+	+			+	+		
	Skin			+	++		+++										++	++			+++
Raccoon dg 4 (Np4)	Liver																+				
	Spleen																+				
	Kidney																+				
	Lung																				
	Small Intestine			+					++								+				
	Large Intestine			++					++								++				
	Mesenteric lymph node																(+)				
	Skin													(+)			+++				

(continued)

Animal	Organ \ Microorganism	No growth	*Acinetobacter* spp.	aerobic bacilli	α-streptococci	*C. perfringens*[1]	*Coryne. auriscanis*[2]	*Enterobacter* spp.	*Enteroc. faecalis*[3]	*Escherichia coli*	*Fungi*[4]	*Lactococcus garviae*	*Lactococcus lactis*	*Macrococcus* spp.	other *Enterobacterales*[5]	*Pseudomonas* spp.	*Psychrobacter* spp.	*S. pseudintermedius*[6]	Coag.-neg. staph.[7]	*Strep. canis*[8]	*Strep. dysgalactiae*	*Vagococcus* spp.
Raccoon dog 5 (Np5)	Liver	+																				
	Spleen												(+)					(+)				
	Kidney						+						(+)					+	(+)	(+)		
	Lung						+						+	(+)	(+)			+	(+)			
	Small Intestine					+					+		+					+	(+)	(+)		
	Large Intestine			+		+++				(+)	+		+					+	+	(+)		
	Mesenteric lymph node						(+)											(+)				
	Inguinal lymph node																	++	(+)	(+)		
	Brain						+											(+)	(+)			
	Reproductive Organs																	(+)				
	Skin						++							++				+++	+			

[1]*C. perfringens*, *Clostridium perfringens*; [2]*Coryne. auriscanis*, *Corynebacterium auriscanis*; [3]*Enteroc. faecalis, Enterococcus faecalis;* [4]Fungi, not further identified; [5] include *Buttiauxella* spp., *Leclercia* spp., *Pantoea* spp., *Rahnella* spp., *Raoultella* spp., *Serratia* spp.; [6]*S. pseudintermedius*, *Staphylococcus pseudintermedius*; [7]Coag.-neg. staph., coagulase negative staphylococci; [8]*Strep. canis*, *Streptococcus canis*

Table S2: Biological data and pathological findings of the five investigated raccoon dogs presented with sarcoptic mange

**Biological data**				**Pathological findings**	
**Animal**	**Sex**	**Age**	**Nutritional status**	**Gross**	**Histopathology**
Raccoon dog 1 (Np1)	M	juvenile	Not assessable	**Skin**: Alopecia with severe lichenification, crust formation and hyperpigmentation of the skin; Ectoparasitosis, mild (ticks and louse flies)	**Skin**: Epidermal hyperplasia and hyperkeratosis, diffuse, severe, with mild eosinophilic dermatitis and numerous intracorneal mites; **Tonsils**: Follicular hyperplasia, mild; **Retropharyngeal lymph node**: Follicular hyperplasia, severe; **Lung:** Bronchopneumonia, necrosuppurative, multifocal,, acute, mild; **Pulmonary lymph node**: Follicular hyperplasia, severe; **Liver**: Hepatolipidosis, diffuse, mild; **Spleen**: Follicular hyperplasia, mild; **Intestine**: Enteritis, lymphocytic, multisegmental, chronic, mild; **Mesenteric lymph node**: Follicular hyperplasia, severe; **Peyer's patches** : Follicular hyperplasia; **Inguinal lymph node**: follicular hyperplasia, severe
Raccoon dog 2 (Np2)	F	adult	Good	**Skin**: Alopecia with severe lichenification, crust formation and hyperpigmentation of the skin; Ectoparasitosis, mild (ticks)<br><br>**Intestine**: Endoparasitosis, mild (nematodes); Catarrhal enteritis, segmental, acute, mild.	**Skin**: Epidermal hyperplasia and hyperkeratosis, diffuse, mild, with mild eosinophilic dermatitis and single intracorneal mites; **Nasal mucosa**: Rhinitis, suppurative and necrotizing, focal, subacute, mild, with intralesional foreign material; **Retropharyngeal lymph node**: Follicular hyperplasia, mild; **Lung**: Pneumonia, bronchointerstitial, lymphohistiocytic, diffuse, chronic, mild, with multifocal involvement of eosinophil granulocytes; airway smooth muscle hypertrophy, diffuse, moderate; **Mesenteric lymph node**: Follicular hyperplasia, severe; **Peyer's patches**: Follicular hyperplasia, severe
Raccoon dog 3 (Np3)	M	juvenile	Poor	**Skin**: Alopecia with severe lichenification, crust formation and hyperpigmentation of the skin; Ectoparasitosis, severe (ticks); **Intestine**: Endoparasitosis, moderate (nematodes); Catarrhal enteritis, segmental, mild	**Skin**: Epidermal hyperplasia and hyperkeratosis, diffuse, severe, with mild eosinophilic dermatitis and numerous intracorneal mites; **Lung**: Bronchopneumonia, suppurative, multifocal, subacute, mild; Airway smooth muscle hypertrophy, diffuse, moderate; **Intestine**: few intraluminal nematodes, **Peyer's patches plates**: Follicular hyperplasia, mild

(continued)

Biological data				Pathological findings	
**Animal**	**Sex**	**Age**	**Nutritional status**	**Gross**	**Histopathology**
Raccoon dog 4 (Np4)	M	juvenile	Not assessable	**Skin**: Alopecia with severe lichenification, crust formation and hyperpigmentation of the skin; Ectoparasitosis, severe (ticks, louse flies; skin scraping during necropsy showed numerous mites (morphologically consistent with *Sarcoptes* sppp.); **Intestine**: Endoparasitosis, mild (nematode); Catarrhal enteritis, segmental, mild	**Skin**: Epidermal hyperplasia and hyperkeratosis, diffuse, severe, with mild eosinophilic dermatitis and numerus intracorneal mites; **Retropharyngeal lymph node**: Follicular hyperplasia, severe; **Lung**: Bronchopneumonia, suppurative, multifocal, subacute, moderate; **Liver**: Hepatitis, necrotizing, (sub)acute, mild; **Spleen**: hyperplasia of the red and white pulp, moderate; **Peyer's patches**: Follicular hyperplasia, severe; **Bone marrow**: Trilinear hyperplasia
Raccoon dog 5 (Np5)	M	adult	Poor	**Skin**: multifocal thinning of the haircoat; Ectoparasitosis, mild (ticks); **Intestine**: Endoparasitosis, mild (cestdodes)	**Skin**: Epidermal hyperplasia and hyperkeratosis, diffuse, severe, with mild eosinophilic dermatitis and numerous intracorneal mites; **Tonsils**: Follicular hyperplasia, moderate; **Retropharyngeal lymph node**: Follicular hyperplasia, mild; **Trachea**: Calcification of the cartilage, mild; **Lung**: Pneumonia, bronchointerstitial, lymphoplasmacytic and eosinophilic, diffuse, chronic, mild, ; **Liver**: Hepatitis, necrotizing, multifocal, acute, moderate; **Stomach**: Follicular gastritis, multifocal, chronic, moderate; **Spleen**: hyperplasia of the red and white pulp, mild; **Intestine**: Enteritis, mostly eosinophilic , segmental, acute, mild; **Kidney**: Fibrosis, interstitial, focal, chronic, severe; **Aortic lymph node**: Follicular hyperplasia, severe; **Inguinal lymph node**: Follicular hyperplasia, severe; **Bone marrow**: Trilinear hyperplasia
